# 离子淌度质谱技术在中药化学成分分析中的研究进展

**DOI:** 10.3724/SP.J.1123.2022.01028

**Published:** 2022-09-08

**Authors:** Rongrong ZHAI, Wen GAO, Mengning LI, Hua YANG

**Affiliations:** 中国药科大学中药学院, 江苏 南京 210009; School of Traditional Chinese Pharmacy, China Pharmaceutical University, Nanjing 210009, China

**Keywords:** 离子淌度质谱, 中药, 化学成分, 综述, ion mobility-mass spectrometry (IM-MS), traditional Chinese medicines (TCMs), chemical components, review

## Abstract

离子淌度质谱(IM-MS)是一种将离子淌度分离与质谱分析相结合的新型分析技术。IM-MS的主要优势不仅是在质谱检测前提供了基于气相离子形状、大小、电荷数等因素的多一维分离,而且能够提供碰撞截面积、漂移时间等质谱信息进而辅助化合物鉴定。近年来,随着IM-MS技术的不断发展,该技术在中药化学成分分析中受到越来越多的关注。首先,IM-MS已成功应用于改善中药复杂成分尤其是同分异构体或等量异位素等成分的分离;其次,IM-MS可通过多重碎裂模式辅助高质量中药小分子质谱信息的获取;此外,IM-MS提供的高维质谱数据信息还可促进中药复杂体系多成分的整合分析。该文在对IM-MS分类和基本原理进行概述的基础上,从分离能力及分离策略、多重碎裂模式、多维质谱数据处理策略3个方面,重点综述了IM-MS在中药化学成分分析中的应用,以期为IM-MS在中药化学成分研究提供参考。

## Abstract (en)

Ion mobility-mass spectrometry (IM-MS) is a combination of ion mobility separation and mass spectrometry technologies. In IM-MS, analytes are ionized by the ion source to form gas-phase ions, which are then rapidly separated using ion mobility based on their mobility difference, under the influence of both neutral buffer gas and an electric field, and then traversed and detected using mass spectrometry, which can separate ions based on mass-to-charge ratio. Furthermore, IM-MS could provide not only mass-to-charge ratio parameters like MS^1^ and MS^2^ spectra but also new structural information for component identification like collision cross-section values, drift time, arrival time, compensation voltage, and so on, which can be employed to resolve complex chemical components, especially indistinguishable isomers using mass spectrometry alone. In recent years, with the development of IM-MS technologies, IM-MS has become more widely employed in chemical analysis in traditional Chinese medicines (TCMs). Firstly, IM-MS was been successfully used in the separation of complex TCMs complex extract from interfering isobaric species. Secondly, IM-MS also offers new types of MS/MS fragmentation modes, and the combination of IM separation and fragmentation modes enables the acquisition of more specific and detailed fragment ion spectra. Thirdly, the collision cross-section is introduced by IM-MS, which is a unique physicochemical property of a component. Related data post-processing strategies based on experimentally derived collision cross-section values have been continuously developed in recent years to make full use of the collision cross-section values, these data post-processing strategies include collision cross-section database matching, theoretical collision cross-section values matching, machine-learning-based collision cross-section values prediction matching, mass-to-charge ratio versus collision cross-section correlation trend lines and so on. In doing so, these diverse strategies can greatly enhance the reliability and accuracy of the structural annotation of TCM compounds. This review primarily briefly introduces the major types and basic principles of IM-MS. The applications of IM-MS in TCM chemical analysis are highlighted in this study. The current applications of IM-MS in improving TCM chemical component separation are summarized, followed by a discussion of several strategies for enhancing separation selectivity. This review also offers some new fragmentation modes, novel data acquisition approaches, and collision cross-section data post-processing strategies applied in TCM qualitative analysis. Finally, the prospect of IM-MS applied in TCM chemical analysis is also discussed. This review provides approaches and ideas for future IM-MS research on TCM’s chemical analysis.

中药作为一种复杂的化学体系,具有结构类型多样、异构现象普遍、成分含量差异大、极性跨度大等分析难点。如何全面和精准解析中药化学成分,进而阐明其发挥药效的物质基础,一直是中药研究中的难点与热点问题之一。离子淌度质谱(ion mobility-mass spectrometry, IM-MS)作为一种新型分析技术,已被广泛应用于脂质组学、代谢组学、蛋白质组学等研究领域^[1⇓⇓-4]^,近年来其在中药化学成分研究中的应用也逐步受到关注。因此,本文重点从离子淌度分离、碎裂模式、数据处理等方面,综述了IM-MS在中药化学成分分析中的应用进展。

## 1 离子淌度质谱概述

IM-MS是一种将离子淌度分离与质谱结合的新型分析技术,最早可追溯到1961年^[5]^。近年来IM-MS技术发展迅速,不同类型的商业化仪器平台相继出现^[6]^,主要有漂移时间离子淌度质谱^[7]^(DTIM-MS)、行波离子淌度质谱^[8]^(TWIM-MS)、高场不对称/差分离子淌度质谱^[9]^(FAIMS-MS/DMS-MS)、捕集离子淌度质谱^[10]^(TIMS-MS)等。目前以DTIM-MS和TWIM-MS在中药复杂化学成分分析中应用较多。

在IM-MS中,待分析物首先经过离子源电离后转化为气相离子,随后气相离子在电场和缓冲气体的相互作用下依据迁移速率不同完成快速预分离,再依据离子的质荷比(*m/z*)进行分离和检测。气相离子的分离既与其本身的形状、大小、所带电荷数等密切相关,也与仪器参数(例如分辨率、温度、电压等)以及缓冲气体的组成等有关^[11]^。与常规质谱相比,IM-MS提供了多一维度的分离,有助于提高分析通量和灵敏度,同时还可进一步结合一维^[12,13]^或二维^[14]^液相色谱(LC、2D-LC)构建三维或四维的分离系统,提升分析性能和效率。在信息参数获取方面,除了提供离子一级质谱信息(MS^1^)、二级质谱信息(MS^2^)和离子丰度等以外,IM-MS还具有离子漂移时间(DT)、到达时间(AT)或补偿电压(CV)等信息。在低电场情况下,还可依据Mason-Schamp方程^[15]^转化计算获得离子的碰撞截面积(CCS),进而提升对离子结构的分析效率。

## 2 离子淌度质谱技术在中药化学成分分析中的应用

### 2.1 分离能力及分离策略

IM-MS的淌度分离能力是对常规色谱-质谱联用的有效补充,特别是在改善异构体或等量异位素的分离方面具有显著优势,一方面在离子淌度分离过程中可增加化合物的分离度,另一方面还可以结合衍生化或者改变漂移气体组成等策略进一步提升化合物分离的选择性。

#### 2.1.1 离子淌度分离

IM-MS增加了新的分离维度,在改善化合物分离方面具有简单、快速的分析优势。例如西红花苷Ⅲ为栀子藏红花素类成分,通常会以顺、反2种异构体形式出现,Wang等^[16]^利用LC-MS分析方法,发现西红花苷Ⅲ在总离子流图中显示为单峰,而利用LC-TWIM-MS技术不仅实现了顺、反西红花苷Ⅲ异构体的快速拆分,还额外发现了新的潜在异构体。Zhang等^[17]^利用2D-LC-TWIM-MS技术分析红参和白参提取物时,实现了10对等量异位素的分离。Bylda等^[18]^利用LC-DMS-MS/MS,在1.5 min内实现了洋地黄毒苷及其3种代谢物的快速分离,与LC-MS/MS相比,该方法空白血样背景噪声降低了10倍。此外,IM-MS还在中药及天然产物小分子位置异构体分离^[19]^、源内异构分析^[20]^、部分手性异构体^[21]^的快速拆分中得以应用。

近年来,一些超高分辨IM-MS,例如环形离子淌度质谱(cIM-MS)^[22]^、无损离子传输结构联用质谱(SLIM IM-MS)^[23]^等也有所发展,能够实现空间结构差异非常微小的化合物分离。Colson等^[24]^利用LC-TWIM-MS(分辨率约40)分析中药娑罗子成分时,发现七叶皂苷1a/1b和异七叶皂苷1a/1b 4个异构体不能被完全分离,4个异构体的AT值分别为9.83、9.75、9.97和9.83 ms,而采用直接注射的超高分辨cIM-MS(分辨率约250)分析时,上述4个异构体的AT值差异增大,显著改善了分离效果。

#### 2.1.2 调整目标离子结构

部分待分析物由于空间结构近似或差异微小,在IM-MS中难以直接达到完全分离,因此也可通过引入金属离子^[25]^、含金属离子手性选择剂(如环糊精类)^[26,27]^或其他衍生化试剂^[28]^改变待分析物离子在气相中的形态、大小等方式提高分离选择性。例如(-)-*α*-红没药醇和(+)-*α*-红没药醇是一对手性对映倍半萜异构体,利用TWIM-MS无法实现直接拆分,有研究报道了引入Ag^+^,增加了样品中*α*-红没药醇对映异构体的分离度^[29]^。Troc等^[30]^等将(-)-表儿茶素和(+)-儿茶素分别与不同的D/L型氨基酸、金属离子、手性冠醚、酒石酸及其组合进行离线衍生化后,再利用TWIM-MS分析发现D-亮氨酸和CuCl_2_的引入可显著改善儿茶素对映异构体的分离度。

#### 2.1.3 调整缓冲气体组成

不同极化率和质量的缓冲气体会显著影响离子-中性分子团簇的形成,进而改变离子迁移率^[31]^。目前最常用的缓冲气体为氮气(N_2_),也可使用氦气、二氧化碳、氩气或混合气体作为缓冲气体,此外还可在缓冲气体中引入气体改性剂(gas modifier)^[32]^、气体掺杂剂(dopants)^[33]^等改善目标离子的分离效率。例如,在FAIMS-MS和DMS-MS技术中常用甲醇、乙腈、异丙醇等气体改性剂来提高分离选择性^[34⇓-36]^。Willems等^[37]^利用FAIMS-MS技术,以N_2_为缓冲气体,比较了甲醇和乙腈2种改性剂对3种单咖啡酰基奎宁酸异构体的分离效果,结果表明甲醇的引入可使3种异构体在1 min内实现基线分离,而乙腈则可能形成多聚体而影响分离效率。Wu等^[38]^采用DMS-MS/MS分析方法,以正丙醇作为缓冲气体(N_2_)改性剂,在8.5 min内实现了3组人参皂苷类成分Rf/Rg_1_/F_11_、Rb_2_/Rb_3_/Rc和Rd/Re的最佳分离与检测。

### 2.2 多重碎裂模式

IM-MS的碎片离子获取方式主要为淌度分离后碎裂(Post-IM),即母离子经过淌度分离后再碎裂,可获得母离子的CCS值、MS^1^、MS^2^和离子丰度等信息。Post-IM方式辅助数据依赖性采集模式(DDA)可通过离子淌度分离,减少共流出成分或背景离子的干扰,获取高质量、高覆盖率的MS^2^数据。例如Wang等^[39]^采用LC-TWIM-MS,以Post-IM方式辅助DDA模式,从防己黄芪汤中鉴定了203种化学成分。而Post-IM方式结合数据非依赖性采集模式(DIA)除了可将复杂样品中所有成分母离子进行离子淌度分离以外,还可根据离子到达时间或漂移时间的信息匹配,实现母离子及其碎片离子的快速关联,提高成分鉴定的准确度与分析效率,其中以TWIM-MS的高清全信息串联质谱采集模式(HDMS^E^)、DTIM-MS的交替图像模式(AF)等较为常见。Feng等^[40]^在对茯苓提取物进行成分表征时,利用LC-TWIM-MS结合HDMS^E^采集模式,在低碰撞能时获取离子一级质谱信息,在高碰撞能时获取碎片离子信息,通过相同保留时间和漂移时间实现母离子及其碎片离子的快速准确匹配,进而根据特征离子和中性丢失等信息对未知化合物进行结构推测。Li等^[41]^利用LC-DTIM-MS结合DIA模式,采用保留时间和漂移时间二维定位策略,从中药附子中快速鉴定了236种二萜生物碱,发现8种潜在的新的酯基取代类型。此外,也有研究者将MS^1^全扫描采集、Post-IM方式辅助DDA采集和HDMS^E^采集等多种模式交替使用以期获得更高的分析效率,应用该组合式质谱扫描方法结合离线二维液相色谱分离技术,从复方丹参滴丸中成功鉴定了403种化学成分^[42]^。

除了Post-IM方式以外,TWIM-MS还具有淌度分离前碎裂(Pre-IM)和时间排列平行碎裂(TAP)。在Pre-IM方式中,母离子经碰撞诱导解离后,再经过离子淌度分离,从而对碎片离子的离子淌度迁移率进行分析,可获取MS^1^、MS^2^、离子丰度、碎片离子CCS值等信息^[43,44]^。Cheng等^[45]^在利用LC-TWIM-MS法分析顺/反双咖啡酰奎宁酸(*m/z*为515.1190)异构体时,发现异构体母离子特征无法区分,而碎片离子(*m/z*为353.0880)则具有不同的AT值,可实现异构体的有效拆分。

TAP方式^[46,47]^可将Post-IM模式与Pre-IM模式相结合,离子在离子淌度分离前进行碎裂获取MS^2^信息,经离子淌度分离后再碎裂得到MS^3^数据,尤其适用于结构相似成分的差异分析。Zhang等^[48]^在利用LC-TWIM-MS技术分析多环多异戊烯基取代间苯三酚类化合物(PPAPs)时,将源内裂解与TAP模式相结合,归纳和总结了4种不同亚型PPAPs的裂解途径和行为,从岭南山竹子中成功鉴定了140种PPAPs。

### 2.3 数据处理策略

高维质谱数据集的整合分析是提升中药小分子化合物鉴定效率的有效手段。IM-MS除了提供离子保留时间、MS^1^、MS^2^、离子丰度等信息以外,还丰富了CCS值、漂移时间等数据类型,尤其是CCS值的引入进一步提升了质谱对于小分子化合物的鉴定能力与效率。

#### 2.3.1 实验碰撞截面积

研究显示化合物的CCS值具有较高的重现性和稳定性^[49,50]^,有研究人员根据对照品测定的实验CCS值构建了一系列本地或共享的CCS数据库,通过检索查询、信息比对,可用于未知化合物的辅助鉴定^[51]^。例如McCullagh等^[52]^利用LC-TWIM-MS自建了甜菊糖苷不同加合物母离子MS^1^、^TW^CCS_N2_数据库(^TW^CCS_N2_:采用TWIM-MS技术和缓冲气体N_2_时所获取的CCS值),通过实测数据与数据库信息的综合比对,有助于提升异构体的鉴定效率,并减少对碎片离子信息的依赖。因此近年来CCS数据库的构建逐步引起了研究人员的关注,例如Picache等^[53]^构建了包含3833个^DT^CCS_N2_值的数据库(^DT^CCS_N2_:采用DTIM-MS技术和缓冲气体N_2_时所获取的CCS值)。采用化学对照品测定方式构建的实验CCS数据库具有较高的准确度,但实验成本相对也较高。

#### 2.3.2 理论碰撞截面积

除了根据化学对照品测定CCS值以外,还可以根据化合物最低能量构型,利用近似投影模型^[54]^、精确硬球散射模型^[55]^、轨迹模型^[56]^等计算模型,计算化合物的理论CCS值。该方法不依赖化学对照品,但计算的要求相对较高。例如姜黄素在溶液中存在酮和烯醇2种互变异构体,Nag等^[57]^运用TWIM-MS检测得到姜黄素酮-烯醇互变异构体的实验^TW^CCS_N2_值,通过密度泛函理论和轨迹模型计算得到姜黄素烯醇的理论CCS值,将两者进行趋势匹配进而成功指认溶液中酮-烯醇互变异构体,进一步通过理论化学发现L形弯曲的酮结构比平面烯醇的空间结构更紧凑,因而姜黄素酮结构的CCS值较小。Wang等^[58]^运用理论CCS值与实验^DT^CCS_N2_值趋势匹配的方法,成功鉴定了4种槲皮素葡萄糖醛酸位置异构体代谢产物。此外,Colby等^[59]^开发了ISiCLE理论CCS值计算方法,创建了开放共享CCS数据库(https://metabolomics.pnnl.gov/),包含了理论CCS值和实验CCS值在内的100多万个CCS数据。

#### 2.3.3 预测碰撞截面积

CCS值通常被认为是化合物的固有性质之一,但已知的CCS值数量有限,在一定程度上限制了其应用。近年来,不断有研究者以已知CCS数据作为训练集,利用支持向量机、人工神经网络^[60]^、偏最小二乘法^[61]^、深度学习^[62]^等机器学习算法,构建CCS预测模型。例如Zhou等采用化合物分子描述符,利用支持向量机模型,先后开发了MetCCS^[63]^、LipidCCS^[64]^、ALLCCS^[65]^等方法实现对代谢物、脂质、小分子化合物等CCS值的大规模预测。化合物CCS预测模型及数据库的构建,极大地拓展了其在中药研究中的应用。Shi等^[66]^分析人参、西洋参、三七的脂质成分时,应用MS-CCS二元搜索鉴定策略,以HMDB/LipidMaps数据库依据MS^1^或MS^2^搜索潜在的化合物结构,以LipidCCS获取潜在的CCS值,进而通过与实验数据匹配,确认未知化合物结构。

#### 2.3.4 质荷比-碰撞截面积趋势分析

不同类型化合物CCS值变化与其*m/z*变化可能呈现一定的规律性,当不同类型的化合物CCS对其*m/z*作回归分析时,会产生各自的回归曲线,该曲线也被称之为CCS-*m/z*趋势线^[67]^。通过化合物趋势线的辅助分析,有助于从复杂体系中快速指认特定类型的化学成分^[68]^。例如Ye等^[69]^分析了山楂中有机酸类、黄酮类、黄烷酮类和三萜类成分的^DT^CCS_N2_与*m/z*函数关系图,结果提示4类化合物的CCS-*m/z*趋势线有显著差异。Li等^[70]^集成利用趋势线分析等方法,发现多电荷原花青素聚合体离子^DT^CCS_N2_与*m/z*为乘幂关系,且与成分聚合度、电荷数、结构单元间A型连接方式正相关。此外,Li等^[71]^还建立CCS-*m/z*乘幂回归趋势线预测区间,结合质量亏损过滤,从味连、云连、雅连、延胡索中分别鉴定出86、102、73和57种异喹啉生物碱类成分,相比于经典的质量亏损过滤策略,该方法的准确度更高。

## 3 总结和展望

综上所述,IM-MS作为新型分析技术,是对常规质谱在中药复杂体系中小分子分离和鉴定方面的有效补充,在中药化学成分分析研究中逐渐被关注。但IM-MS在中药研究中的应用尚处于起步阶段,还有一些值得深入研究的方向和挑战。例如现有IM-MS技术在中药化学成分分析中的研究主要集中在体外分离和表征分析中,很少应用于中药体内代谢产物鉴定、中药代谢组学等研究领域。此外,CCS作为化合物离子的固有性质,可用于中药化学成分的表征,但现有报道的中药化学成分及天然产物CCS非常有限,进一步融合计算机化学、化学计量学、生物信息学等多学科手段来构建中药化学成分及天然产物CCS库,更有助于实现中药化学成分全面、快速、精准分析。
